# Drug-Induced Thrombotic Microangiopathy Arising During the Treatment of Anal Carcinoma After the Use of Mitomycin C

**DOI:** 10.7759/cureus.81731

**Published:** 2025-04-04

**Authors:** Krishna Sheth, Cody Lee, Mihir Patel, Hannah Mathew, Ajith Saju, Sergio Obligado

**Affiliations:** 1 Internal Medicine, Garnet Health Medical Center, Middletown, USA; 2 Internal Medicine, Touro College of Osteopathic Medicine, Middletown, USA; 3 Emergency Medicine, University of Texas Southwestern Medical Center, Dallas, USA

**Keywords:** anal cancer, capecitabine, drug-induced thrombotic microangiopathy, mitomycin, mitomycin c (mmc)

## Abstract

Anal cancer is a rare disease where malignant cells originate in the tissues of the anal canal. This form of cancer is classically treated with a combination of radiation therapy and a chemotherapy regimen that includes mitomycin C. This case illustrates an unusual presentation of thrombotic microangiopathy associated with mitomycin C. A 57-year-old woman with a history of anal carcinoma treated with capecitabine/mitomycin C and radiation was sent to the emergency department by her oncologist for an incidental finding of worsening kidney function noted on a complete metabolic panel done prior to getting radiographic imaging. The patient was admitted to the hospital for suspected acute kidney injury from suspected ureteral obstruction and stent occlusion; however, despite reversal of the stents, renal function did not improve. Renal biopsy confirmed thrombotic microangiopathy and diagnosis of drug-induced thrombotic microangiopathy. This case discusses a side effect of thrombotic microangiopathy from mitomycin C and successful treatment with eculizumab.

## Introduction

Anal cancer is a rare tumor that comprises only 2.5% of all digestive malignancies in the United States [1]. The incidence of new cases of anal cancer in the United States according to the National Cancer Institute’s Surveillance, Epidemiology, and End Results Program (SEER) is 8200 cases per year [1].

Mitomycin C is a quinine antineoplastic that inhibits DNA synthesis and causes tissue injury via direct toxicity of the vascular endothelium [2]. Anal cancer is classically treated with a combination of radiation therapy and chemotherapy, commonly capecitabine and mitomycin C. Mitomycin C can cause irreversible deterioration of kidney function, proteinuria, and hematuria via the hemolytic uremic syndrome (HUS), a type of thrombotic microangiopathy (TMA) [3]. Mitomycin C is one of the better-described chemotherapeutics associated with thrombotic microangiopathy, and its toxicity occurs in a dose-dependent manner [2].

HUS is a rare and serious disease that is defined as a thrombotic microangiopathy. Defining clinical features of HUS include thrombocytopenia, microangiopathic hemolytic anemia, and acute kidney injury (AKI). The incidence of HUS is relatively rare; therefore, accurate epidemiology of the disease is difficult to obtain. The incidence of HUS in 2016 was about 1.2 cases per 100,000 people per year [4]. Underreporting of the disease worldwide skews the actual prevalence of the disease, therefore impacting overall patient care and treatment.

Drug-induced TMA (DITMA) presents similarly to HUS; common presentations include hypertension, subnephrotic proteinuria, renal insufficiency, microangiopathic hemolytic anemia, and thrombocytopenia. Severe complications can arise from DITMA due to microthrombosis and mortality has been reported as high as 75% in some patients with mitomycin-associated TMA. In this report, we describe a patient with DITMA that responded well to eculizumab.

## Case presentation

A 57-year-old woman with a history of anal carcinoma, migraines, radiation cystitis, Hashimoto’s disease, and osteoporosis presented to the Emergency Department for worsening kidney function as noted by her oncologist. The patient received a chemotherapy regimen of capecitabine and mitomycin c for metastatic anal carcinoma a few months prior to the emergency department visit, which resulted in multifactorial anemia. Recent oncology follow-up regarding anal carcinoma showed cancer in remission. Patient's home medications included famotidine 20mg, subcutaneous heparin 5000 units twice daily, and levothyroxine 175 mcg. The patient was an active smoker with 0.5 pack per day for 30 years. Renal ultrasound in the emergency department revealed no masses, cysts, calcifications, or hydronephrosis.

Initial hemoglobin and hematocrit were 7.6 g/dL (reference range: 13.3-17) and 22.8% (reference range: 40.3-50.3%), respectively. Platelets were 184,000 µL (reference range: 132-337 X10^3 µL). Urinalysis was positive for 3+ blood, RBC 43 million/μL, and hyaline casts. The protein/creatinine ratio was 0.86 g/g (reference value: <0.2). The basic metabolic panel showed CO2 of 14 mEq/L (reference range: 22-30 mEq/L), blood urea nitrogen of 34 mg/dL (reference range: 9-20 mg/dL), creatinine of 2.21 mg/dL (reference range: 0.66-1.25 mg/dL), and eGFR of 25.4 mL/min/1.73 m^2^ (reference range: >60). C4 and C3 complement levels were within normal limits. LDH was 383 U/L (reference range: 87-241 U/L) but peripheral blood smear was negative for schistocytes. Her prior creatinine was 0.7 mg/dl three months earlier. On physical exam, the patient was normotensive and had moist mucous membranes, a regular heart rate, and no peripheral edema. She was admitted for AKI with unknown etiology, metabolic acidosis secondary to AKI, anemia, and microscopic hematuria of unknown origin. Non-contrast CT of the abdomen and pelvis revealed no signs of hydronephrosis or genitourinary obstruction.

There remained concerns about the possibility of retroperitoneal fibrosis given her history of radiation treatment. MRI of the abdomen confirmed no presence of hydronephrosis or retroperitoneal fibrosis, but contrast could not be safely given due to her history of AKI. The decision was made by urology to perform cystoscopy and bilateral ureteral stent placement given the possibility of urinary obstruction. Throughout the hospitalization, the patient’s creatinine remained around 2. She was discharged with instructions to follow up with urology as an outpatient for bilateral ureteral stent removal. Renal biopsy was discussed at that time, however not performed due to improvement of renal function.

**Figure 1:**
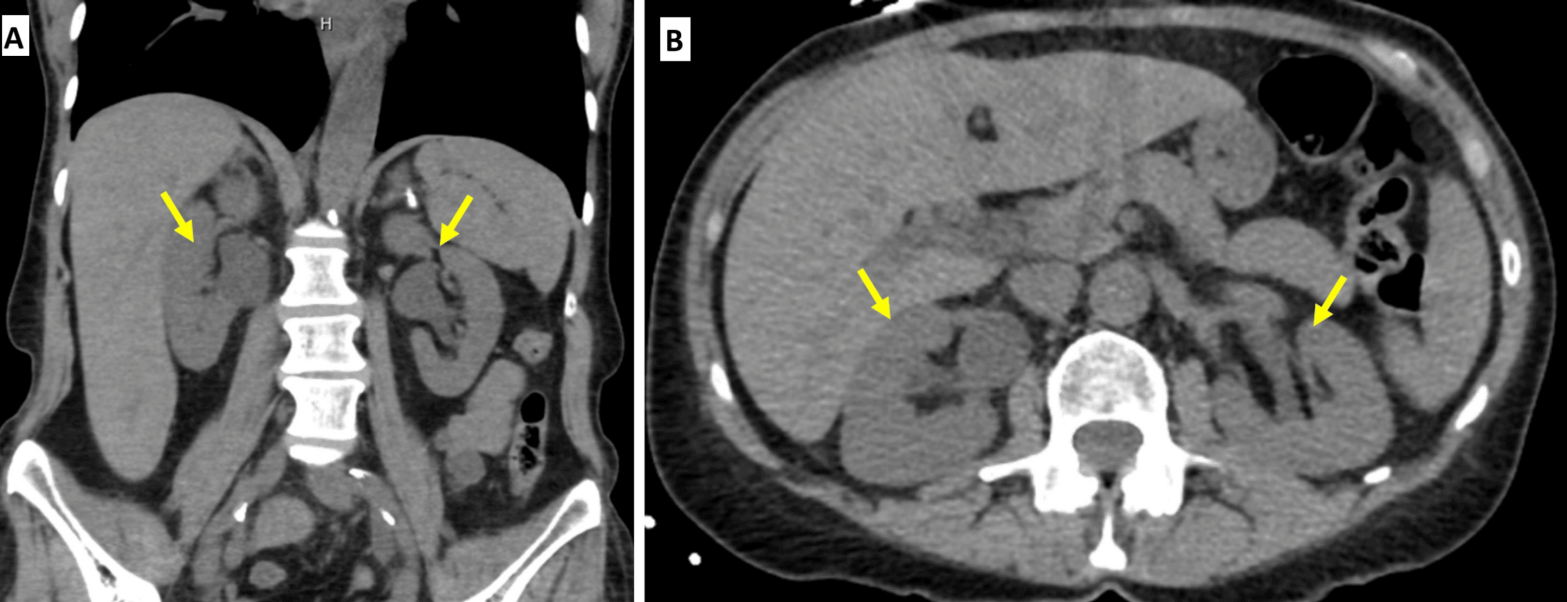
CT abdomen and pelvis show bilateral hydroureteronephrosis (yellow arrows).

One week after initial discharge, on follow-up laboratory testing, creatinine had again increased from 2.0 mg/dL to as high as 3.8 mg/dL. The patient also had new complaints of intractable nausea and vomiting and bilateral flank pain. New-onset hydronephrosis was noted on CT abdomen and pelvis following ureteral stent removal, as seen in Figure 1.

**Figure 2:**
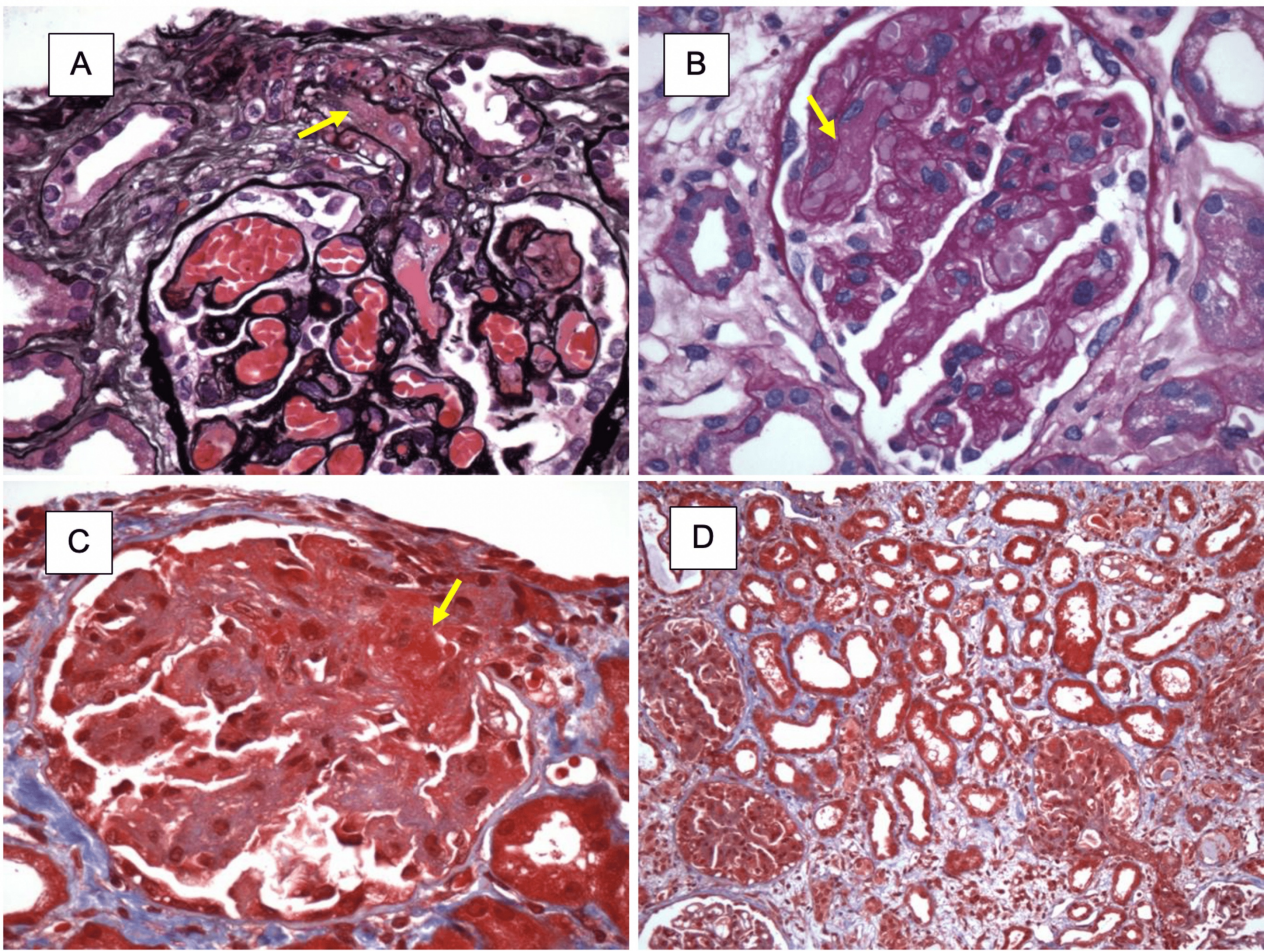
Immunohistological stains of renal biopsy: A: A glomerulus showing RBC congestion and the afferent arteriole contains a fibrin thrombus (arrow) (Jones Methenamine stain, original magnification 60X). B: A glomerulus shows RBC congestion and fibrin thrombus (arrow) (Periodic acid-Schiff stain, original magnification 60x). C: A glomerulus shows global attenuation of capillary lumina and fibrin thrombus (arrow) in the hilar region (Trichrome stain, original magnification 60x). D: Patchy interstitial edema and inflammation are seen. Proximal tubules show simplification and loss of apical brush border (acute tubular injury) (Courtesy of Dr. Stokes at Columbia University, permission was obtained prior to inclusion).

This new worsening AKI was thought to be due to reflex anuria secondary to ureteral spasm following stent placement or acute interstitial nephritis. The patient was readmitted to the hospital due to acute chronic kidney disease of unknown etiology. Her renal function did partially recover, back to a creatinine of 2.0 mg/dL, and renal ultrasound confirmed resolution of hydronephrosis. However, since her creatinine did not return to her prior baseline of 0.7 mg/dl and unexplained anemia persisted, a renal biopsy was performed. Renal biopsy revealed intracapillary and subendothelial electron-dense deposits consistent with (i) thrombotic microangiopathy, acute and subacute, predominantly involving glomeruli, due to HUS, complement-mediated microangiopathic process, (ii) acute tubular injury, patchy with focal RBC casts, (iii) moderate interstitial inflammation and edema, and (iv) mild to moderate arteriosclerosis and arteriolosclerosis. Immunohistochemical stains of the renal biopsy are shown in Figure 2.

ADAMTS13 activity was 71%. Genetic testing for complement gene variants was negative (testing done via Natera; genes tested were ADAMTS13, C3, CFH, CFI, PLG, MMACHC, and DGKE). Nephrology and oncology treated her presumed DITMA with eculizumab 900 mg/week. She was started on penicillin prophylaxis due to risk of Neisseria infection. After consistent treatment with eculizumab, patient’s creatinine had decreased to as low as 1.16 mg/dL and anemia has improved.

## Discussion

TMA is a clinical syndrome that shares the common clinical presentation of microscopic hemolytic anemia, thrombocytopenia, and AKI. Etiologies are diverse and include primary causes (thrombotic thrombocytopenia and atypical HUS) and secondary causes such as preeclampsia, malignant hypertension, HUS, and DITMA [5]. Over the years, the classification of TMA has evolved as our understanding of the underlying molecular mechanisms behind them has improved. The term HUS is now typically reserved for Shiga toxin-mediated hemolytic uremic syndrome [5]. Thrombotic thrombocytopenia is now usually reserved for thrombotic microangiopathies that are characterized by ADAMTS-13 deficiency. The syndrome which was previously referred to as “atypical HUS”, is due to various etiologies that are now better understood as complement-mediated thrombotic microangiopathy (CMTMA) [5]. In these disorders, either genetic variants in complement or acquired antibodies against complement factor H initiate the vascular injury that leads to the clinical syndrome. In CMTMA diseases, uncontrolled activation of the alternative complement pathway causes deposition of complement attack complexes that cause microvascular injury in tissues [5]. DITMA can be either due to antibodies against tissue that stimulate complement activation or via non-immune mechanisms whereby the specific drug causes direct tissue injury and hence complement activation [5].

Quinine was one of the first drugs described as causing DITMA; the mechanism has been well characterized since that time. Until the FDA started regulating quinine use in the community, it was one of the most common causes of DITMA [6]. The mechanism of quinine-induced TMA is due to drug-dependent antibody binding to the glycoprotein IIb/IIIa epitope on platelets and causing cell injury [7]. Many other drugs have been described as causing DITMA, such as calcineurin inhibitors, antibiotics (trimethoprim-sulfamethoxazole), and gemcitabine, just to name a few. Gemcitabine is a chemotherapy frequently associated with DITMA. One case series described its frequency as 1% of patients receiving gemcitabine (three patients out of 264 patients) [8]. 

Mitomycin, which is the drug of interest in our case series, has been reported as a cause of TMA in several case studies and retrospective studies. In a meta-analysis of 344 studies reporting DITMA, mitomycin was considered a potential cause of TMA in 54 patients [9]. It can be difficult to assess the chemotherapy definitively responsible for TMA since multiple agents are typically used in combination. Typically, mitomycin is administered with 5-FU in these studies; 5-FU has never been associated with TMA so it can be presumed that mitomycin is the causative agent in these studies. The mechanism of mitomycin-associated TMA is not clear; whether it is immunologically mediated or due to direct vascular injury is unknown. Most reports seem to indicate that mitomycin-induced TMA is likely dose-related toxicity [10]. The toxicity of mitomycin tends to be correlated with the total dose received; some reports suggest that a total cumulative dose of 60 mg carries a higher risk of developing TMA [11]. TMA from mitomycin typically occurs within 4-6 weeks of the dose, but cases have been described as far out as six months from treatment [12]. 

In our case, it seems likely that mitomycin is the drug responsible for the patient’s DITMA. The diagnosis was not straightforward due to the lack of thrombocytopenia and lack of significant schistocytes on the smear. This delay in diagnosis was unfortunate and led to unnecessary cystoscopy and stent placement. The decision was made to treat our patient with eculizumab, with an excellent response. Eculizumab, a recombinant monoclonal antibody, may be useful for DITMA as it binds to complement C5-inhibitor and blocks the generation of proinflammatory C5a and C5b-9. It has been shown to improve outcomes in thrombotic microangiopathy due to atypical HUS (CMTMA) [13]. Case reports have described success with the use of eculizumab for the treatment of TMA induced by bleomycin, gentamycin, and oxaliplatin. In addition, eculizumab was used in a case study of a breast cancer patient who developed TMA after treatment with mitomycin-C [14]. Another complement blocker, ravulizumab, has been used successfully in a case of gemcitabine-associated TMA [15]. Other potential treatments for TMA such as glucocorticoids and therapeutic plasmapheresis have not been as successful for DITMA. For now, evidence for the treatment of DITMA with complement blockers will have to rely on case studies and retrospective studies.

## Conclusions

DITMA is a well-described type of secondary HUS that can lead to severe complications including renal failure. Mitomycin C is a commonly used chemotherapeutic treatment that is associated with TMA. Treatment currently is not well defined. However, eculizumab, a terminal complement inhibitor, has shown promise for the treatment of DITMA. It should be considered for patients who develop TMA after mitomycin C treatment.
